# Computer Vision-Assisted Semiautomatic Analysis of Zooplankton in a Longitudinal Study of the Ecological State of Lake Baikal

**DOI:** 10.3390/biology15090695

**Published:** 2026-04-29

**Authors:** Olga Olegovna Rusanovskaya, Sergey Sergeevich Oreshkov, Anastasiya Andreevna Demidova, Taysia Pavlovna Rzhepka, Eugene Anatolyevich Silow, Nickolai Vasilyevich Shadrin, Svetlana Vladimirovna Shimaraeva, Maxim Anatolyevich Timofeyev

**Affiliations:** 1 Institute of Lake Baikal Biology, Irkutsk State University, Irkutsk 664003, Russia; dem.anastasia@bio.isu.ru (A.A.D.); tasyarjpk@bio.isu.ru (T.P.R.); silow@bio.isu.ru (E.A.S.); shimaraeva@gmail.com (S.V.S.); director@bio.isu.ru (M.A.T.); 2 MaritimeAI, Kaliningrad 236000, Russia; sergey.oreshkov@maritimeai.net; 3 A.O. Kovalevsky Institute of Biology of Southern Seas of RAS, Sevastopol 299011, Russia; shadrin@ibss-ras.ru

**Keywords:** Lake Baikal, zooplankton, deep neural networks, metric learning, human-in-the-loop, computer vision

## Abstract

Studying zooplankton in freshwater ecosystems is crucial for ecological research, providing insight into ecosystem health, biodiversity, and water quality. This study focuses on developing a hybrid approach for studying and analyzing zooplankton communities using machine learning and human expert analysis. The goal of the study was to automate the labor-intensive process of zooplankton analysis as part of a long-term Lake Baikal monitoring program (since 1945), while maintaining continuity with traditional methods. A software and algorithmic system were developed to automate the analysis: images were processed using a two-stage pipeline (object detection using YOLO V11, classification using metric learning and visual transformers), and complex cases and new species were sent to specialists for verification. Over 240,000 images from 811 samples were processed, and models are updated using verified data to adapt to seasonal changes. An updatable database of labeled zooplankton images suitable for statistical analysis and research has been created. A comparison of manual and machine analysis revealed no significant differences in species composition, with accurate detection in 87% of images. This approach allows for scalable monitoring and the accumulation of labeled data arrays for the development of computer vision methods and the assessment of the state of Lake Baikal’s ecosystem.

## Simple Summary

Zooplankton is an important component of aquatic ecosystems. The analysis of its taxonomic structure and quantitative indicators helps to identify the dynamics of ecosystem changes. The program of ecological monitoring of the zooplankton community of Lake Baikal has been implemented since 1945. This article introduces a novel hybrid method for researching zooplankton in Lake Baikal. It combines machine learning algorithms with expert insights. Microscopic images of plankton are processed using an algorithm that detects, classifies, and quantifies the abundance of organisms. As a result, a database was created and carefully selected, periodically updated, made up of images of organisms living in Lake Baikal. This database serves as a source of environmental statistics and can be used for further research in the fields of computer vision, microscopy, and ecology.

## 1. Introduction

The project of long-term environmental monitoring of Lake Baikal “Point No. 1” has been implemented at the Institute of Biology of the ISU since February 1945 initiated by M. Kozhov [1,2,3] and is included in the Russian Book of Records as the longest project of regular environmental monitoring in the history of science [4].

The founder of the project is an outstanding researcher of Lake Baikal Professor Mikhail Mikhailovich Kozhov. The project is based on an assessment of the state of both phytoplankton and zooplankton, since planktonic organisms are the foundation of the ecosystem functioning of any large lake, and their monitoring allows forecasting of global changes occurring on our planet [5,6]. During the project (80 years), approximately 31,000 samples were collected from different water layers. A total of 12,000 samples were used to assess the species composition and abundance of zooplankton, and 19,000 samples were used to analyze phytoplankton. The results of their processing were compiled into a database containing more than 7.5 million records. The samples are processed by microscopy, enabling us to evaluate the species composition and population density of all planktonic organisms. This is a time- and labor-intensive process performed by highly qualified specialists. The introduction of computer image processing allows us to raise the project to a new technical level while maintaining continuity.

### Alternative Approaches

Since surveying microorganisms in aquatic habitats is not a new task, several alternative methods for automating zooplankton identification have been described in the literature. These methods vary in imaging techniques, feature extraction methods, and classification models. Most rely on a water flow through a chamber equipped with a high-resolution camera that captures images for later recognition. The chamber is often integrated into submersible equipment that directs water flow inside.

DeepLO [7] was created to facilitate analysis of images obtained from the in situ LOKI system, which captures digital images by concentrating zooplankton in a chamber equipped with a digital camera. DeepLOKI utilizes a deep neural network based on ResNet18 architecture, achieving an accuracy of 89% on the dataset with 33 different categories composed of 194,479 images. The system also includes utilities for data storage during cruises with limited access to the internet and an extensive user interface facilitating its usage; however, it was designed to identify a list of key species and does not imply extension of that list.

A similar approach was used by [8] with an in situ camera that is deployed to monitor lake zooplankton. In contrast, this approach utilizes an ensemble of several neural networks trained on a significantly smaller dataset of 17,943 manually labeled images of 35 categories.

The approach described in [9] is most similar to ours—it also utilizes several principles of the metric learning approach, and it also implements a human-in-the-loop approach, creating a system for assisted image annotation. This approach relies more on the K-Means algorithm and a set of hand-picked features that describe object clusters. As a result of testing on the WHO dataset with a whole set of species, the algorithm achieved an accuracy of 79%. This system is also designed for the setting of a camera observing the flow of water with a plankton inside it.

The previously widely used systems, including ZooScan and VPR, are largely based on developments from 2010 or earlier [10]. While these platforms have made important contributions, they were not designed with the flexibility required to accommodate continuous changes in sample composition and throughput typical of long-term monitoring programs. Furthermore, many existing systems rely on towed submersible platforms, which differ substantially from the historical sampling methodology used in our study and are therefore not directly applicable [11,12,13].

Although several approaches have been developed, they focus mainly on in situ recognition. In our case, as samples are collected and analyzed separately, it was important not to alter an already well-established process but rather to inject additional capabilities into the analysis process.

## 2. Materials and Methods

### 2.1. Samples Collection

Plankton samples for the study were collected at one stationary point (“Point No. 1”) located in the open part of Southern Baikal, at a distance of 2.7 km from the shore (51°52′48″ N; 105°05′02″ E) above a depth of about 800 m across from the biostation of the Institute of Lake Baikal Biology at Irkutsk State University (Bolshie Koty settlement).

Plankton was collected weekly throughout the year, with time of melting and freezing of ice, when the sampling point was not accessible by ship nor on foot.

Zooplankton were sampled with a Juday plankton net with an inlet diameter of 37.5 cm and a mesh size of 0.099 mm (Marneo, St. Petersburg, Russia). The layer of 0–250 m was separated into the following fractions: 0–10, 10–25, 25–50, 50–100, 100–150 and 150–250 m. The samples were fixed with a 4% formalin solution and allowed to settle for 3 weeks, after which the sediment was concentrated and examined in a counting chamber via a light microscope [14]. The total number of zooplankton species was estimated to be thousands of specimens/m^2^. The significance of differences in zooplankton abundance between the study methods was determined using the Mann–Whitney criterion, while it was assumed that there were no significant differences between the samples at *p* ≥ 0.05. The coefficients were calculated in the R programming environment (R version 4.5.1).

Microscopy-assisted manual laboratory processing was used to determine the species composition and abundance of each sample. Subsequently, the samples were photographed and uploaded to the automated sample analysis system. Following image analysis of the captured objects, the system generated aggregated statistics on species composition and abundance while retaining the same format of data collection.

### 2.2. Sample Analysis System

For image processing and storage, we designed and implemented an automatic sample analysis system, which handles storage of sample images, detects objects on images, classifies known types of objects, and detects novel ones.

The system implements a human-in-the-loop paradigm for image processing. Images are first processed through an automated computer vision pipeline, after which predictions are reviewed and verified by experts. Since the introduction of the automated sample analysis system, all samples have been digitized by photographing individual microscopy-identified objects.

### 2.3. Image Processing Pipeline

**Figure 1:**
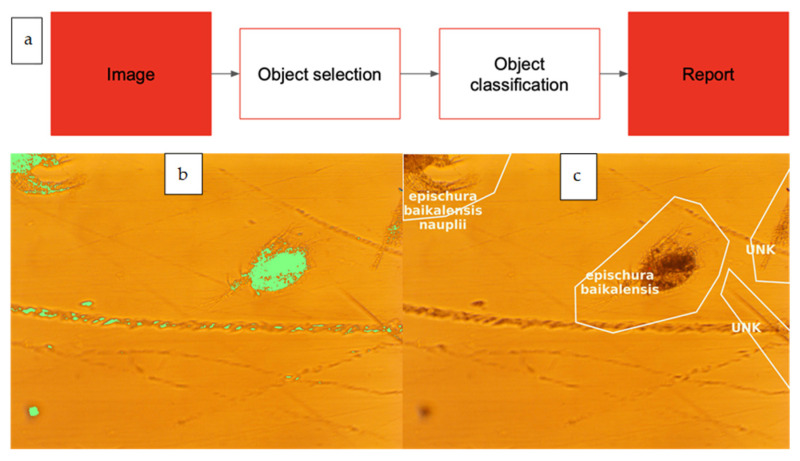
Two-step image processing. (**a**) Overview of process. (**b**) Detection of individual objects is performed by Yolo V11 network architecture, highlighting parts of the image that could be an object. (**c**) Classification is made by a separate algorithm, which labels each recognized object with a known class or dedicated “unknown” label.

The computer vision pipeline processes images with a two-step process (Figure 1), which includes object detection to detect zooplankton organisms and image classification to classify detected entities.

Since the beginning of the implementation of the project in November of 2021, all acquired samples have been processed with a sample analysis system, and all objects found in samples are being photographed and stored.

To acquire an initial dataset suitable for training computer vision models, manual object detection and manual object classification by human experts were required.

To acquire the initial dataset for computer vision, experts manually processed 12,174 images taken from routinely gathered samples, in a two-step process of object selection with the following classification. These images were fully manually annotated and remain part of routine observations and part of initial project.

**Figure 2:**
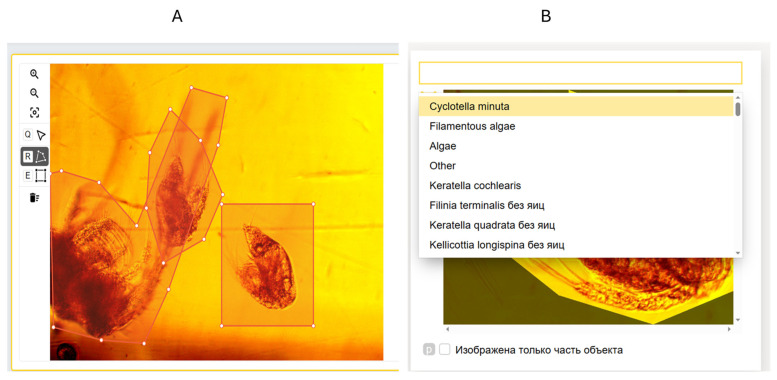
The stages of the manual sample processing process: (**A**) selecting objects; (**B**) classification of objects (*Filinia terminalis* без яиц—*Filinia terminalis* without eggs; *Keratella quadrata* без яиц—*Keratella quadrata* without eggs; *Kellicottia longispina* без яиц—*Kellicottia longispina* without eggs; Изoбражена тoлькo часть oбъекта—only part of the object is depicted).

Manual object selection is composed of two substages (Figure 2), namely, object selection and selection verification, to minimize the probability of incorrect image annotation. The first part, object selection itself, can be distributed between several executors; however, during the second part, the supervisor evaluates images with selected objects.

The second step is an object classification, which means that a human expert identifies an object from the object image acquired during the first step and assigns a suitable class to it.

### 2.4. Computer Vision Assisted Processing

As soon as the first processed samples were received, we started implementation of computer vision algorithms to assist human experts and gradually took the majority of the work from human experts.

**Figure 3:**
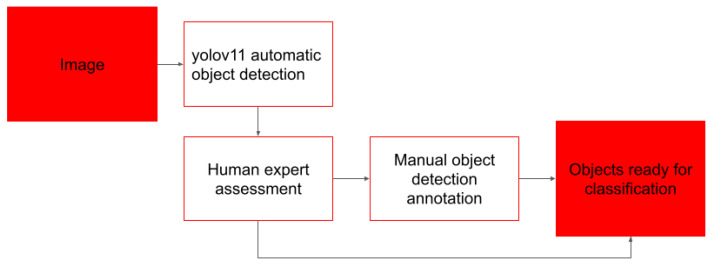
Design of object detection implementation. After detection of individual objects, individual object detections with estimated probability below threshold of 0.8 are passed for manual evaluation.

The computer vision algorithm processes all images, after that, a human expert evaluates the quality of that object detection. Only images that are evaluated as too difficult for the algorithms undergo manual processing (Figure 3).

We ameliorated both steps of the original image processing routine with computer vision algorithms and designed its integration in the way that algorithms take care about of the bulk of images and objects and leave only difficult or unknown objects for human processing.

During the first step, an algorithm based on Yolo V11 [15] architecture detects individual objects from the image. If the human supervisor deems the result of that object detection as incorrect, the image is then processed by humans. The automatic object detector is capable of correctly processing 87% of images containing a single object or set of visibly decoupled objects. The main obstacles for computer vision algorithms we encounter are overlaps between objects and physical artifacts of the background, such as scratches on the surface of a counting chamber, and several overlapping objects. Input images were resized to 640 × 640 pixels, and built-in data augmentation strategies (including scaling, flipping, and mosaic augmentation) were applied automatically during training. The optimization process jointly minimized bounding box regression, objectness, and classification losses. The model achieved a mean Average Precision (mAP) of 0.685 computed over IoU thresholds ranging from 0.5 to 0.95, and an mAP of 0.903 at IoU = 0.5, demonstrating robust detection and localization performance after training on a subset of 30,150 images with hand-annotated objects. As object detection step is used without classification, all objects in training dataset for detector are labeled with a single class of “object”.

### 2.5. Classification with Metric Learning

**Figure 4:**
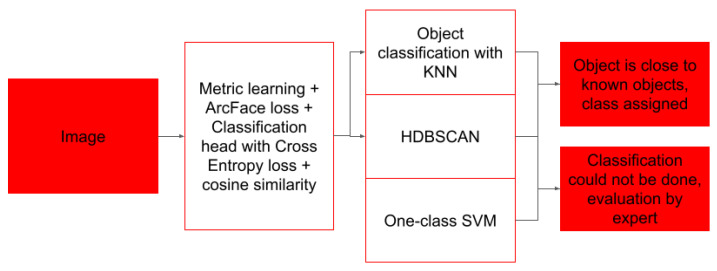
Overview of image classification algorithm. Image of individual object detected by detection algorithm or specified by human is passed to the embedding neural network, which produces compressed embedding vector of size 128. This representation vector is then compared against vectors of known objects by KNN algorithm, picking major object class from objects with similar vectors. Number of heuristics estimating distance, vector density and novelty of embedding vector is applied—One-class SVM estimates how studied vector is close to nearby vectors, while HDBSCAN evaluates local density of vectors describing similar objects.

To classify objects from the initial image, we utilize the metric learning approach. A neural network with a visual transformer architercture ViT_b_16 [16] as a feature extractor and an embedding generator with an output size of 128 values was trained via the ArcFace loss function [17], and cosine similarity was used as a measure of the difference between the embeddings of different classes [18] using AdamW optimizer with an initial learning rate of 3 × 10^−4^ and a weight decay of 1 × 10^−5^ with batch size of 32 samples. A classification head was appended to the embedding network during training to enhance the discriminative capability of the learned representations (Figure 4).

For this layer, a supplementary cross-entropy loss function was applied. Finally, output vectors from the embedding layer are evaluated on the basis of proximity of images of various classes with the KNN algorithm.

### 2.6. Outlier Detection in Metric Space

**Figure 5:**
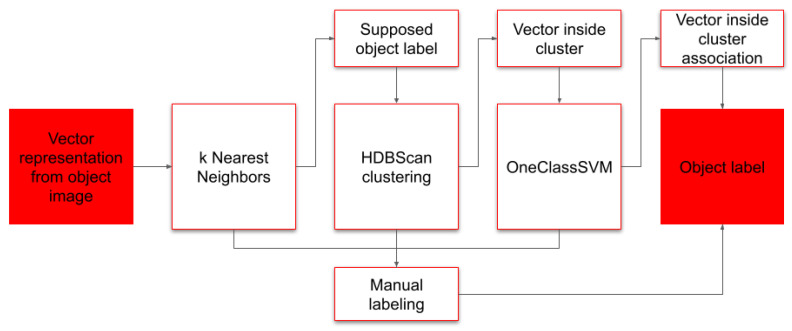
Processing workflow of object vector representations.

Once vector representations for objects were obtained, we store them as the Faiss [19] flat index to facilitate subsequent lookup operations (Figure 5). As we utilized cosine similarity for vector representation mining, they tended to form clusters in the embedding space. To explore these structures, we utilized the OneClassSVM [20], k-Nearest Neighbors (KNN) and HDBScan algorithms [21]. KNN is used to determine the distance to the closest objects of various classes. For objects of novel types, it provides information on possible similarities with already known object classes. Here, we utilize a basic heuristic—if a novel object vector is situated among at least 5 object vectors of the same type *a* and distance from these objects is lower than average distance between objects of that type *a*, we decide to label that novel object with that type *a*. Next, we utilize HDBScan clusterization of objects for type *a*—we assess if a novel object falls into any cluster formed by objects of type *a*. If novel objects do not fall into any HDB cluster, we assume that the object could be of unknown class or of an unusual form of known class. Images of such objects are sent to human experts for verification. If a novel object falls into the HDB cluster, we fit the OneClassSVM model for objects belonging to that cluster. OneClassSVM certainty prediction below the threshold of 0.95 is considered a novel object and routed to be evaluated by human experts.

The vector representation gets a supposed label by nearest-neighbor analysis of already known objects, then it must pass two additional filters. The first filter is based on HDBScan, which accepts only labels for objects falling into clusters formed by objects of the same label. The second filter is a certainty prediction based on OneClassSVM which is fitted to all objects of that same label.

### 2.7. Models Adaptation

Both detection and classification steps of the computer vision pipeline are being updated through frequent retraining following an active learning design pattern utilizing constantly growing curated datasets.

**Figure 6:**
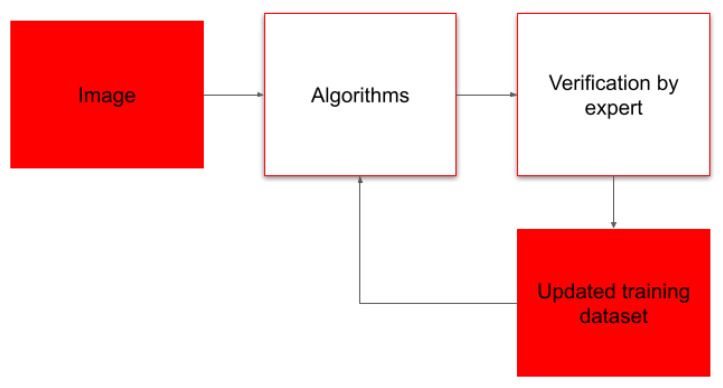
Continuous model update workflow. For training datasets we use only images verified by human experts.

As new water samples are constantly being added into the system, we continuously update the models to accommodate emerging species, variability of object images, and data drift while updating training datasets with verified object images and annotations as new samples are being processed (Figure 6).

Object detection and metric learning algorithms are updated multiple times throughout the year to account for seasonal variation (e.g., winter–summer differences) and other ecological changes. The training dataset is constructed exclusively from images validated by human experts to prevent the incorporation of algorithm-induced biases. The main training criteria for each model are to achieve improvement of classification and detection metrics—IoU and mAP with expert-verified images. Model adaptation and retraining occur after verification of images and objects marked as difficult or unknown for classification algorithms or after sample processing is finished, ensuring that pipeline algorithms will be capable of handling challenging objects in the future.

## 3. Results

**Figure 7:**
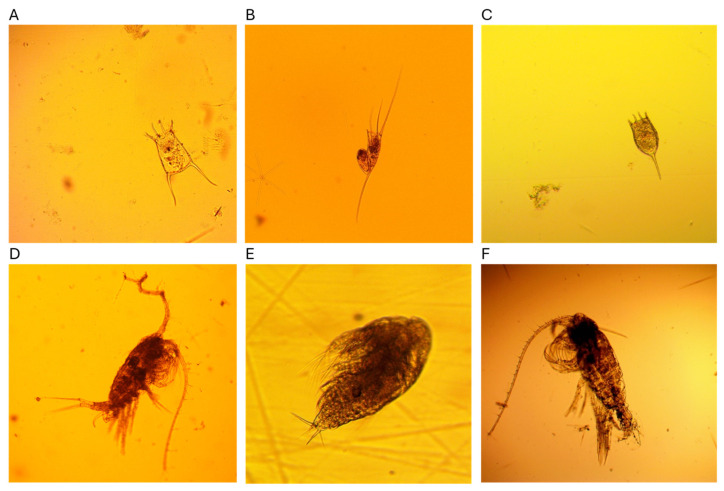
Representatives of the zooplankton community: (**A**) *Keratella quadrata*, (**B**) *Kellicottia longispina*, (**C**) *Keratella cochlearis*, (**D**) *Epischura baikalensis* male, (**E**) *Epischura baikalensis* nauplial stage, (**F**) *Epischura baikalensis* female.

The ecological monitoring of Lake Baikal is based on the assessment of the species composition and abundance of the plankton community. The community of Baikal plankton, like in other lakes, consists of phyto-, zoo-, and bacterioplankton. The net zooplankton (Figure 7) is represented by crustaceans from the orders Copepoda (*Epischura baikalensis* Sars, 1900; *Cyclops kolensis* Lilljeborg, 1901; *Harpacticella inopinata* Sars, 1908), Cladocera (*Daphnia longispina* Müller, 1776; *Bosmina longirostris* Müller, 1785), and rotifera. Rotifers are represented year-round by *Kellicottia longispina* Kellicot, 1879; *Keratella quadrata* Müller, 1786; *Keratella cochlearis* Gosse, 1851; and *Filinia terminalis* Plate, 1886. Winter–spring forms include *Synchaeta pachypoda* Jaschnov, 1922; *Synchaeta pachypoida* Kutikova et Vassiljeva, 1982; *Synchaeta prominula* Kutikova et Vassiljeva, 1982; *Synchaeta* sp.; *Notholca grandis* Voronkov, 1917; *Notholca intermedia* Voronkov, 1917; and *Collotheca* sp. Summer–autumn forms include *Asplanchna priodonta priodonta* Gosse, 1850; *Asplanchna herricki* Guerne, 1888; *Bipalpus hudsoni* Imhof, 1891; *Collotheca mutabilis* Hudson, 1885; *Conochilus unicornis* Rousselet, 1829; *Euchlanis dilatata* Ehrenberg, 1832; *Gastropus stylifer* Imhof, 1891; *Lecane luna* Müller, 1776; *Polyarthra vulgaris* Carlin, 1943; *Synchaeta* sp.; *Synchaeta pectinata* Ehrenberg, 1832; *Synchaeta oblonga* Ehrenberg, 1832; *Synchaeta grandis* Zacharias, 1983; *Synchaeta stylata* Wierzejski, 1893; and *Trichocerca (s. str.) capucina* Wierzejski et Zacharias, 1893.

**Figure 8:**
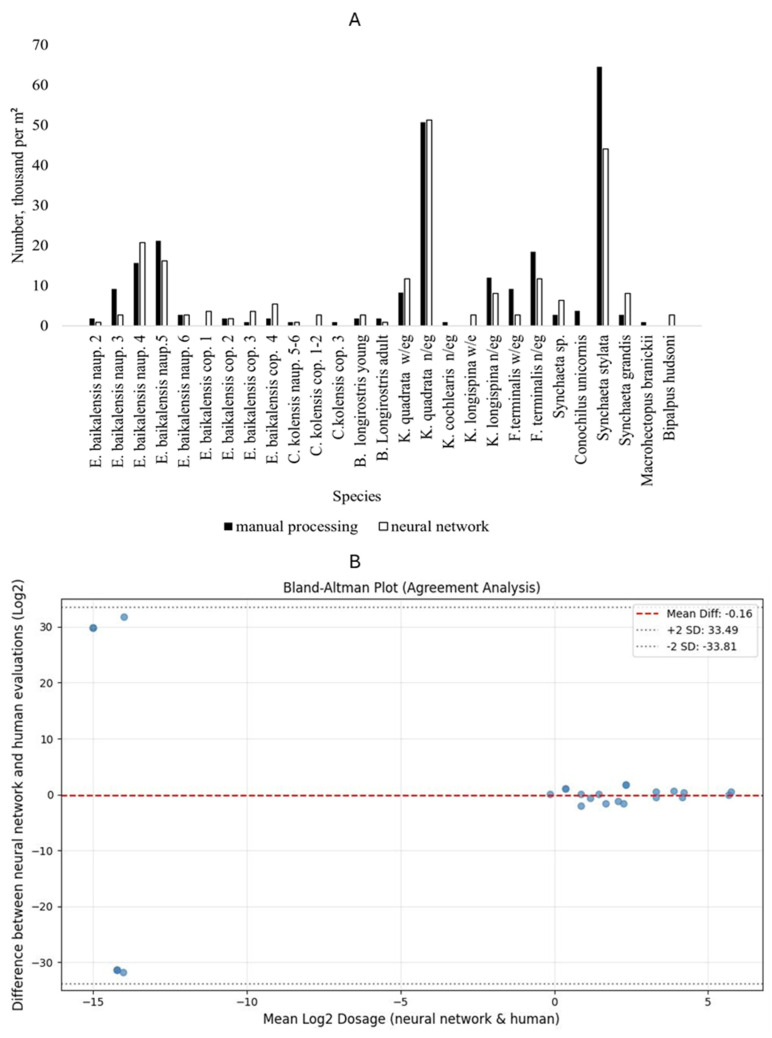
(**A**) Example of the results of microscopy and machine processing of samples from the pelagic zone of Southern Baikal in a layer of 0–50 m at Point No. 1. (**B**) Bland–Altman agreement analysis on species concentration in two dimensions. Each dot in plot represents difference between estimations by expert and algorithm. *X* axis represents mean of log2 evaluations, *Y* axis represents difference between log2 of evaluations. Dotted lines represent levels of 2 standard deviations from mean value.

In five hydrobiological samples, taken during the summer–autumn period from a 0–10 m layer, species diversity and abundance were analyzed using microscopy and machine-based processing. Since the selected samples had no significant differences, the results of one summer sample are presented here (Figure 8).

Thirteen species of organisms were encountered in this sample, which were classified into certain categories based on their characteristics. Five nauplial and four copepodite stages were identified for *E. baikalensis*; nauplial and copepodite stages were also identified for *C. kolensis*; *B. longirostris* was represented by juveniles and adults; rotifers were divided into individuals with eggs (w/eg) and individuals without eggs (n/eg).

The analysis of the results showed (Figure 8) that average zooplankton abundance obtained by manual processing of zooplankton samples was 3.50 ± 8.59 thousand specimens/m^2^ (N ± SD). When using a neural network, the average zooplankton abundance was 1.18 ± 2.90 thousand specimens/m^2^ (N ± SD). A pairwise permutation test applied to the L1 distance between human and algorithmic estimations yielded a *p*-value of 0.573, indicating no statistically significant difference between the two sets of estimates. Furthermore, Wilcoxon test does not show enough differences between human expert and computer vision estimations with *p*-value 0.6446, and Pearson correlation between estimations r = 0.94 Additionally, Bland–Blatman plot analysis does not reveal any significant discrepancies.

In 30% of cases, there are no differences in species abundance between manual and machine processing. In 40%, the neural network did not count some organisms, most often representatives of the Rotifera type, which can overlap each other in the images. In 30%, the neural network showed higher numbers than with manual processing, mainly representatives of the species *Epischura baikalensis* Sars, 1900.

In general, there are no differences in species abundance between manual and machine sample processing—the neural network determines all the species in the sample. The differences are noted only in numerical indicators, with absolute number of deviations not exceeding variations emerging between repeated evaluations of the same sample.

**Figure 9:**
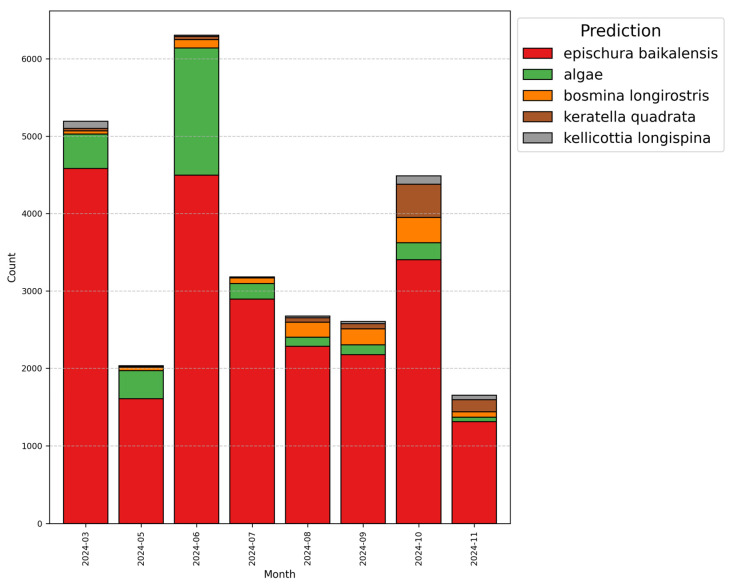
Characterization of species abundance detected from march till November of year 2024. Top 5 species, including algae presented.

Automated image-based analysis of zooplankton samples facilitates discovery of distinct temporal patterns in species composition and relative abundance (Figure 9).

For instance, over the year of 2024 there were a total of 132 samples, collected in March, and from May till November 31,812 images were analyzed. Throughout the study period among automatically processed objects, *E. baikalensis* remained most abundant, comprising 79% of total detections on average, with a peak of 85% in July of 2024. *B. longirostris* accounted for 4% of processed objects, showing maximum abundance in September with 7.67%, while *K. quadrata* abundance grew from 1 to 2% in spring and summer up to 9% in October and November of 2024. Machine learning algorithms also estimate numbers of questionable recognitions, leaving them for expert validation. After validation is complete, computer vision algorithms undergo additional training to obtain new knowledge and assembled recognition data becomes available for any kind of statistical analysis.

## 4. Discussion

From the perspective of computer vision systems design, we selected a human-in-the-loop approach with active learning as an approach to update computer vision algorithms. The usual approach is to create a classical classification algorithm that works on a predefined set of species, detecting and classifying only those.

Our goal was to create a system incorporated into the daily researcher workflow. This goal poses several major challenges: robustness to new incoming samples; adaptability to changing environmental conditions reflected in sample composition and appearance; and the ability to detect previously unobserved species and present them to human experts. Additionally, the system should minimize incorrect classifications to maintain the overall quality of work.

To achieve this, we introduced an additional step into the workflow: saving sample images. While this increases the total processing time, it allows the task to be distributed among several experts, since the image capture does not require extensive species classification expertise. Subsequent processing can then be performed through image analysis, which is also amenable to parallelization.

This modification also enabled routine dataset aggregation, allowing for the collection of datasets large enough to support modern computer vision algorithms. After compiling the first manually labeled dataset, we implemented a computer vision pipeline and deployed it as an expert assistant. Sample images are first processed automatically; then, only the hard and confusing images, as well as those likely containing novel species, are transferred to human experts for manual evaluation. As of December of 2025, the computer vision system handles the storage and processing of more than 240,000 images from 811 samples, accumulated starting in November of 2021.

A downside of this approach is its vulnerability to data shifts: when the input data changes significantly, the performance of the computer vision algorithm suffers, and the system reverts to fully manual operation. We observe this during spring periods, for example, when the abundance of algae makes them shift from rare objects to obstacles for object recognition.

Across multiple models trained on different dataset subsets, we observed high variability in classification quality, ranging from 0.05 mAP@5 during training of classifiers on their first samples collected during algae bloom season to 0.61 mAP@5 after classifier trained of samples collected over 3 years of observations. While object detection is validated by human experts and is correct in 77.1% of images, classification remains a bottleneck. To address this, we developed heuristics that compare new incoming images against training data. These heuristics allow for automatic processing of a portion of the dataset without human intervention, ranging from 15% to 63%, depending on seasonal complexity, with summer images being more difficult due to algae interference.

As machine learning algorithms are prone to some error rate, risk of mislabeling of potion of objects persists. This issue is addressed first by active learning design—algorithms are being frequently retrained on objects, labeled as unknown by previous version of pipeline. The second approach is eventual validation of already processed data, performed by data curation experts. While previous manual workflow relied on manual labor of experts, it was not possible to review sample evaluation without full reanalysis of sample; therefore, error rate of manual workflow remains largely unknown.

Another promising but unexplored direction is scalability. The revised workflow enables the inclusion of third-party human experts, fostering collaborative identification of rare species. This framework improves identification accuracy and promotes broader scientific collaboration.

Furthermore, as the computer vision system currently processes images obtained from single origin, we expect it to be heavily overfitted for samples from Baikal Lake. We expect that fine-tuning is necessary to scale system workflow on other laboratories and water reservoirs in order to maintain quality of models work.

Finally, improved data transparency not only benefits ecological research but also contributes to public understanding of ecosystem dynamics. Open datasets generated through this process support innovation in both ecological science and computer vision, serving as critical resources for developing and refining automated species identification tools.

## 5. Conclusions

The developed system can become an important element of the Baikal monitoring program “Point No. 1”, necessary to estimate and forecast the ecological state of the lake, the impact of climate change on its ecosystem, to monitor biological safety and the spread of invasive alien species, as well as a tool for detecting new species.

## Data Availability

The dataset of images and annotations fully verified by experts during the current study are available in the GitHub repository at https://zenodo.org/records/18769622?token=eyJhbGciOiJIUzUxMiJ9.eyJpZCI6IjBjYzgwZGI1LTNjZDctNDgxYS05ZDk0LTU5ZTQzNjkwNThhYyIsImRhdGEiOnt9LCJyYW5kb20iOiI1NTg1MDBjMjkxZmY5OWUxMDg4NDJjMjc0ZGJkMjU3NyJ9.Z6415riGhbNeo2spr4VgswUFFe55SmxPxLYNlxDvFK1FAMYVf1aZJ7eC94JiTjxpEF9pLDrW9uNLmxdUOmmhqg, accessed on 25 February 2026.
